# Quality of life/spirituality, religion and personal beliefs of adult and elderly chronic kidney patients under hemodialysis^1^

**DOI:** 10.1590/0104-1169.3595.2495

**Published:** 2014

**Authors:** Suzana Gabriela Rusa, Gabriele Ibanhes Peripato, Sofia Cristina Iost Pavarini, Keika Inouye, Marisa Silvana Zazzetta, Fabiana de Souza Orlandi

**Affiliations:** 2 Undergraduate student in Gerontology, Universidade Federal de São Carlos, São Carlos, SP, Brazil; 3 PhD, Associate Professor, Universidade Federal de São Carlos, São Carlos, SP, Brazil; 4 PhD, Adjunct Professor, Universidade Federal de São Carlos, São Carlos, SP, Brazil

**Keywords:** Renal Insufficiency, Chronic, Renal Dialysis, Quality of Life, Spirituality, Adult Health, Health of the Elderly

## Abstract

**OBJECTIVE:**

to assess the quality of life of chronic kidney patients undergoing hemodialysis, using the WHOQOL-bref and WHOQOL-SRPB.

**METHOD:**

a descriptive and cross-sectional study was undertaken at a kidney replacement therapy service in the interior of the state of SP. The 110subjects who complied with the inclusion criteria answered the Subject Characterization Instrument, the WHOQOL-bref and WHOQOL-SRPB.

**RESULTS:**

most of the respondents were male (67.27%), with a mean age of 55.65 years, Catholic (55.45%), with unfinished primary education (33.64%) and without formal occupation (79.08%). The WHOQOL-bref domains with the highest and lowest mean score were, respectively, "psychological" (µ=74.20) and "physical" (µ=61.14). The WHOQOL-SRPB domains with the highest and lowest mean score were, respectively, "completeness and integration" (µ=4.00) and "faith" (µ=4.40).

**CONCLUSIONS:**

the respondents showed high quality of life scores, specifically in the dimensions related to spirituality, religion and personal beliefs. Losses were evidenced in the physical domain of quality of life, possibly due to the changes resulting from the chronic kidney disease and hemodialysis treatment.

## Introduction

Among the different diseases in this current epidemiological scenario with a predominance of chronic non-transmissible diseases, one of the conditions that increasingly stands out is Chronic Kidney Failure (CKF), due to the gradual increase in the prevalence and incidence of this disease all over the world and its high morbidity and mortality, thus representing a great social and economic challenge for public health in the global context^(^ 1 ^-^ 2 ^)^.

CKF involves a renal injury and the progressive and irreversible loss of kidney functions (glomerular, tubular and endocrine). In its most advanced phase (called end-stage CKF), the kidneys are no longer able to maintain the normality of the patient's internal environment and dialysis treatment is needed^(^ 3 ^)^.

Terminal CKF and its treatments can influence the patients' biological, psychological, economic and social dimensions and interfere in their Quality of Life (QoL). In addition, chronic kidney patients tend to have a lower QoL, a characteristic already associated with the growing population of CKF patients^(^ 4 ^-^ 5 ^)^.

In this context, scientific studies that mainly focu on the most compromised aspects of the QoL in this patient group are increasingly necessary to guide interventions aimed at improving the level of health in this population^(^ 6 ^)^.

Religion and spirituality are important for dialysis patients, as they are influential in important aspects of QoL and in coping with the disease^(^ 2 ^)^. Associations have been demonstrated between greater religiousness and/or spirituality and a better quality of life^(^ 2 ^,^ 7 ^-^ 8 ^)^. Hence, the assessment of these patients' QoL with regard to spirituality and religion is an essential step to raise awareness on the importance of these factors in this population's life and wellbeing, as well as to promote actions that help the patients in their contact with the disease, its treatments and its harmful aspects in different contexts of daily life, and the WHOQOL-Spirituality, Religion and Personal Beliefs (SRPB) is an effective and innovative tool for that end, as this generic instrument enhances the construct created for the WHOQOL-100 and WHOQOL-bref^(^ 9 ^)^, created to measure the QoL with regard to spirituality, religion and personal beliefs^(^ 7 ^)^.

Recently, the Brazilian version of this instrument has been validated, and its study revealed the satisfactory psychometric properties of the WHOQOL-SRPB in Brazilian Portuguese^(^ 10 ^)^.

It should be highlighted that, until date, no Brazilian studies have been published that used this instrument for QoL assessment with regard to the spirituality, religion and personal beliefs of a CKF population.

The objective in this study was to assess the QoL/spirituality, religion and personal beliefs of chronic kidney patients undergoing hemodialysis through the WHOQOL-bref and WHOQOL-SRPB.

## Method

A descriptive and cross-sectional study with a quantitative approach was developed at a renal replacement therapy service in the interior of the State of São Paulo - Brazil.

The sample consisted of people with the following inclusion criteria: age of 18 years or older, medical diagnosis of CKF, undergoing outpatient hemodialysis at the aforementioned service. The participants were selected by convenience and according to their availability to collect the data. The first patients who agreed to participate were interviewed, until reaching the number needed to compose the sample (N=110).

The sample size was statistically determined to estimate the mean quality of life domain scores in the WHOQOL-SRPB, with a significance level of 1% (alpha or type I error), and a sampling error of 10%, 7% and 5% of the general mean global SRBP score (d=0.4, d=0.3 and d=0.2) (Table 1).

**Table 1 -:** Calculation of sample size for means estimation of quality of life domains of WHOQOL-SRPB*

Quality of life domain	Mean	Standard deviation	Sample size d=0.4	Sample size d=0.3	Sample size d=0.2
Connectedness to spiritual being/force	4.21	0.83	28	51	110
Meaning of life	4.14	0.70	21	37	82
Awe	4.23	0.65	18	32	71
Completeness and Integration	4.00	0.69	20	36	79
Spiritual strength	4.18	0.80	27	48	107
Inner peace	4.07	0.64	17	31	68
Hope and Optimism	4.18	0.66	19	33	73
Faith	4.40	0.50	11	19	42
Global SRPB	4.18	0.52	12	20	45

* Calculations considering alpha at 1%, estimated mean and standard deviation of current sample corresponding to N=110 patients and sampling error of d=0.4, d=0.3 and d=0.2, according to Hulley and Cummings (1988) and Fonseca and Martins (1994).

Based on the results, it was verified that, in view of the mean and standard deviation of the quality of life domain scores in the current sample and a sampling error of d=0.2, the minimal number of subjects was 110 patients with a view to a representative sample for all domains and the global WHOQOL-SRPB score.

The data were collected between January and April 2013. The contact with the study participants took place at a private room inside the outpatient clinic. The objective and other information about the research were presented and possible doubts were clarified. After the subjects had consented, the signing of the Informed Consent Form was requested. Next, an interview was held, during which the instrument to characterize the subjects was applied, as well as the QoL assessment instruments of the World Health Organization (WHO), WHOQOL-bref and WHOQOL-SRPB.

The instrument used to characterize the subjects was specifically elaborated for this research, including questions on the subject's identification data (name, age, sex), sociodemographic data (marital situation, education, per capita income, questions on religion and personal beliefs) and clinical information (length of hemodialysis).

WHO's WHOQOL Group^(^ 11 ^)^ developed the WHOQOL-bref, which has been validated in Brazil^(^ 12 ^)^. It includes four domains: physical, psychological, social relations and environment. In this study, the score from 0 to 100 was used and, the higher the score, the better the QoL.

The Mental Health Division of the World Health Organization (WHO) started developing the WHOQOL-SRPB module in the mid-1990's^(^ 9 ^)^. In Brazil, the version of the WHOQOL-SRPB was validated in 2011^(^ 10 ^)^. The instrument consists of 32 items, distributed in eight facets (Connectedness to a Spiritual Being or Force, Meaning of Life, Awe, Wholeness and Integration, Spiritual Strength, Inner Peace, Hope and Optimism and Faith)^(^ 10 ^)^. The mean final facet and global scores can range from 1 to 5 and, the higher the score, the better the individual's QoL.

It should be highlighted that, when using modules from the WHOQOL-Group instruments, the generic instrument (WHOQOL-100 or WHQOOL-bref) is used in combination^(^ 10 ^)^. Therefore, in this study, the application of the WHOQOL-bref and WHOQOL-SRPB were chosen.

The data collected through the interviews with the subjects were transported to a worksheet in the software Excel for Windows 2010. Next, using the statistical software IBM SPSS(r) (Statistical Package for the Social Sciences) version 19.0, the descriptive analysis was elaborated with the help of frequency tables, position measures - mean, median, minimum and maximum - and dispersion (standard deviation). Cronbach's alpha coefficient was calculated to check the internal consistency of the WHOQOL-bref and WHOQOL-SRPB questionnaires. A Cronbach's alpha coefficient ≥ 0.70 was considered satisfactory^(^ 10 ^-^ 12 ^)^.

Approval for the project was obtained from the Research Ethics Committee at Universidade Federal de São Carlos - UFSCar, under opinion No. 165/2012.

## Results

Table 2 shows the subjects' sociodemographic variables and clinical categories. Among the 110 subjects assessed, the majority was male (67.27%), with unfinished primary education (33.64%), with a partner (63.64%), living in São Carlos (76.36%), with 1 to 3 persons at home (62.73%) and without formal employment (79.08%). As regards the religious belief, 55.45% were catholic. Concerning the self-reported level of religiousness, the majority self-identified as "highly/extremely religious" (66.28%). Most of the participants considered they were practitioners in their respective religious communities (67.27%).

**Table 2 -:** Description of sociodemographic and clinical categorical variables of 110 subjects studied. São Carlos, SP, Brazil, 2013

Variable	Category	N	%
Age (years)			
	31 to 59	66	60.00
	60 or more	44	40.00
Sex			
	Male	74	67.27
	Female	36	32.72
Marital situation			
	With partner	70	63.64
	Without partner	40	36.36
Education			
	Illiterate	11	10.00
	Unfinished primary education	37	33.64
	Finished primary education	17	15.45
	Unfinished secondary education	13	11.82
	Finished secondary education	21	19.10
	Unfinished higher education	2	1.81
	Finished higher education	9	8.18
Religious beliefs			
	Catholic	61	55.45
	Evangelical	31	28.19
	Others	18	16.36
Religiousness			
	Nothing	7	6.36
	Little	7	6.36
	Moderate	23	20.91
	A lot	52	47.27
	Extremely	21	19.01
Practitioner			
	Yes	74	67.27
	No	36	32.72

As regards the analyses of the sociodemographic and numerical clinical variables, the respondents' mean age was 55.65 years, (Q_2 _= 57, SD = 12.87, *x* *_min _*= 31, *x* *_max _*= 85). The mean declared per capita income was 888.58 *reais* (Q_2_ = 539.58, SD = 1151.20, *x* *_min _*= 77.75, *x* *_max _*= 7500.00) and the mean length of HD was 46.35 months (Q_2_ = 36, SD = 47.69, *x* *_min _*=0.06, *x* *_max _*= 240).

Table 3 shows the respondents' mean scores on the WHOQOL-bref. The highest scores were found in the domains "psychological" and "social relations", while the "physical" domain obtained the lowest mean score.

**Table 3 -:** WHOQOL-bref domain scores for 110 subjects studied. São Carlos, SP, Brazil, 2013

Domains	Mean	Standard deviation	Median	Variation observed	Cronbach’s alpha
Physical	61.14	18.54	60.71	11-100	0.75
Psychological	74.20	15.12	75.00	17-100	0.72
Social Relations	73.11	19.11	75.00	17-100	0.70
Environment	67.67	15.59	67.19	25-100	0.78

As regards the internal consistency of the WHOQOL-bref, the instrument showed satisfactory reliability for the global questionnaire (α=0.83). For the domains, Cronbach's alpha ranged between 0.70 and 0.78 (Table 3).

Concerning the internal consistency of the WHOQOL-SRPB, an excellent Cronbach's alpha is observed (0.95). In Table 4, it is verified that the subjects' mean scores on the WHOQOL-SRPB were high, per domain as well as globally, indicating a good QoL, specifically for the questions related to spirituality, religiousness and personal beliefs. It should be highlighted that the domain "Faith" obtained the highest mean score (μ=4.40).

**Table 4 -:** WHOQOL-SRPB domain scores for 110 subjects studied. São Carlos, SP, Brazil, 2013

Domains	Mean	Standard deviation	Median	Variation observed	Cronbach’s alpha
Connectedness to spiritual being/force	4.21	0.83	4.13	1.0-5.0	0.92
Meaning of life	4.14	0.70	4.00	1.0-5.0	0.85
Awe	4.23	0.65	4.25	2.5-5.0	0.70
Wholeness and Integration	4.00	0.69	4.00	1.0-5.0	0.77
Spiritual strength	4.18	0.80	4.25	1.0-5.0	0.88
Inner peace	4.07	0.64	4.00	2.0-5.0	0.83
Hope and Optimism	4.18	0.66	4.25	1.8-5.0	0.77
Faith	4.40	0.50	4.25	3.0-5.0	0.88
Global SRPB	4.18	0.52	4.17	2.7-5.0	0.85

## Discussion

In this study, most of the interviewed subjects were adults. In a study involving data from chronic kidney patients under dialysis treatment registered at the Nephrology Unit of the Hospital de Base em São José do Rio Preto (SP), aimed at characterizing the patients; verifying the causes of CKF; identifying the diseases associated with CKF and surveying the treatment type and the patients' current access, the sample included more adult than elderly people (77%)^(^ 13 ^)^. Nevertheless, a trend exists towards a gradual increase in the number of elderly patients with CKF, as advanced age can be considered an influential factor in the increase in the number of elderly HD patients^(^ 14 ^)^.

Most of the respondents were male (67.27%). According to the 2011 Brazilian Dialysis Census^(^ 15 ^)^, among 50,128 dialysis patients, 57.3% were men. In a study that involved patients from 12 dialysis services in the city of Belo Horizonte (MG), aimed at identifying factors associated with the health-related quality of life of elderly hemodialysis patients, it was verified that 56.5% of the respondents were male^(^ 6 ^)^.

The subjects' mean age in this study was 55.65 years. In a study at a hemodialysis service of the Hospital de Base de São José do Rio Preto, aimed at assessing the QoL of CKF patients receiving hemodialysis treatment and identifying the daily activities that can compromise their QoL, the subjects mean age was 53.1 years^(^ 1 ^)^.

As regards the education level, individuals with unfinished primary education were predominant (33.64%). In that research, the authors also verified that 64.8% of the subjects had not finished primary education^(^ 1 ^)^.

The mean per capita income the participants declared was 888.58 *reais*. A study undertaken at an outpatient clinic for hypertensive patients in Campina Grande (PR), aimed at studying the initial stage of CKF in this population, showed that the subjects' mean per capita income was also relatively low and corresponded to less than one minimum wage for 75% of the sample^(^ 16 ^)^.

The number of respondents who reported having a fixed partner was higher than those declared without partners/alone, as well as in the study developed at 12 dialysis services in the city of Belo Horizonte (MG), which also used a sample of chronic kidney patients in HD^(^ 6 ^)^.

The predominant religion among the participants was Catholicism. Other studies support this result, like the study undertaken at a general philanthropic hospital in a city in the state of Minas Gerais, in which the objective was to identify and clinically validate the defining characteristics proposed for the nursing diagnosis "impaired spirituality", with CKF patients receiving HD, in which the population declared Catholic was 79.2%^(^ 17 ^)^.

The interviewees' mean length of HD in this research was 46.35 months. In the study undertaken at the Hospital de Base de São José do Rio Preto (SP), aimed at assessing the QoL of people undergoing HD treatment, it was observed that the mean length of treatment in the sample was 28.5 months, ranging between 1 and 108 months^(^ 1 ^)^.

The mean scores obtained when applying the WHOQOL-bref in the sample of the 110 subjects showed that the "physical" domain was more affected. In the search for research that used the WHOQOL-bref to measure the QoL of CKF patients, several studies were found. Different studies developed with the CKF population found the lowest mean score in the physical domain^(^ 8 ^,^ 18 ^)^.

The low mean scores in the physical domain reaffirm the harmful aspects of CKF and hemodialysis treatment for the patients' QoL, with regard to their wellbeing and physical health^(^ 19 ^)^.

The WHOQOL-bref domains with the highest scores in this study were "psychological" (m=74.20; SD=15.12) and "social relations" (m=73.11; SD=19.11). Similar results were found in different studies published in the Brazilian and international literature involving the CKF population receiving dialysis^(^ 8 ^,^ 18 ^)^.

Concerning the assessment of the respondents' QoL in this study, using the WHOQOL-SRPB, high mean scores were found, ranging from 4.00 (SD=0.69) in the domain "wholeness and integration" to 4.40 points (SD=0.50) in "faith".

The domain "faith" refers to the comfort and wellbeing faith offers to the individuals, positively influencing their way of life, while the domain "wholeness and integration" refers to the feeling of balance among mind, body and soul, and how it can influence the harmony among actions, thoughts and feelings^(^ 20 ^)^.

The global internal consistency of the WHOQOL-SRPB applied to the 110 subjects in this study was 0.95, similar to what was found in the validation study of that instrument in the Brazilian context, in which the consistency coefficient was 0.96^(^ 10 ^)^, and also similar to the coefficient found in the study about the validation of the WHOQOL-SRPB in French, equal to 0.96^(^ 21 ^)^.

In another study that explores the relation between spirituality and QoL and investigates the contribution of spirituality in other QoL domains, involving 103 schizophrenic patients from an Outpatient Service of the PGIMER Institute in India, when applying the WHOQOL-SRPB, the authors obtained the highest mean score in the domains "spiritual connectedness" and "faith", both scoring 3.4. The domain with the lowest mean score was related to "awe" (m=3.2)^(^ 22 ^)^.

In a Brazilian study, the authors used the WHOQOL-SRPBi (scale of importance granted to the SRBP facets) to assess the importance both samples of subjects, with and without chronic illness, granted to the facets of the WHOQOL-SRPB, besides associating their QoL with the presence of a chronic illness and with the importance granted to the domains. According to the results obtained through the application of the WHOQOL-100, the authors identified that the QoL domain - SRPB - obtained a slightly higher mean score than the other domains for the chronic patients, but that this difference was statistically insignificant. The WHOQOL-SRPBi score also revealed that the patients obtained a higher mean score than the healthy individuals, highlighting that they granted more importance to the aspects related to spirituality, religion and personal beliefs^(^ 23 ^)^.

In the validation study of the French version of the WHOQOL-SRPB, 561 voluntary participants completed the instrument. According to the mean scores obtained, the domains with the highest and lowest mean score were, respectively, "Awe" (μ=3.84) and "Faith" (μ=2.43), while the global domain obtained a mean score of 3.05^(^ 21 ^)^. In comparison with the present study, differences are observed in the results, as all domains of the WHOQOL-SRPB showed high mean scores, particularly "Faith", with the highest mean score (4.40), and the global domain with a mean score of 4.18. The difference between the results obtained in this research and the abovementioned results can be explained by the cultural differences between the populations: while the Brazilian population is mostly acknowledged and culturally religious, the populations in countries like France and Switzerland have a secular culture and high prevalence of agnostic and atheist individuals^(^ 21 ^,^ 24 ^)^.

Finally, it should be observed that this research is limited by the analysis of patients from only one dialysis center in the interior of the state of São Paulo, which makes it impossible to generalize the results to other contexts. In addition, the WHOQOL-SRPB was validated recently and, therefore, there are no Brazilian and international studies about spirituality, religion and personal beliefs of the CKF population under hemodialysis that could enrich the discussion of the results.

## Conclusion

In view of the objective proposed in this study, it was concluded that the "physical" domain of the WHOQOL-bref revealed the lowest mean scores for the research subjects, showing greater physical commitment and low QoL in this group. Through the application of the WHOQOL-SRPB, it was observed that the chronic kidney patients obtained higher QoL scores, specifically in the dimensions related to Spirituality, Religion and Personal Belies. Among these, "Faith" and "Awe" were the highest.

Possible practical applications of these research results are the health professionals' awareness raising about QoL, specifically focusing on spirituality, religion and personal beliefs as important factors, which should be considered and respected during care delivery. These factors can serve as important tools in coping with CKF and renal replacement therapy, and the professionals can discuss them simultaneously with therapeutic work as a strategy to offer comfort, tranquility and wellbeing to the patient.
